# Efficacy of National Nosocomial Infection Surveillance score, acute-phase proteins, and interleukin-6 for predicting postoperative infections following major gastrointestinal surgery

**DOI:** 10.1590/S1516-31802007000100007

**Published:** 2007-01-04

**Authors:** José Eduardo de Aguilar-Nascimento, Jair Gimenez Marra, Natasha Slhessarenko, Cor Jesus Fernandes Fontes

**Keywords:** C-reactive protein, IL-6, Albumin, Transferrin, Prealbumin, Infection, Surgery, Proteína C-reativa, IL-6, Albumina, Transferrina, Infecção, Cirurgia

## Abstract

**CONTEXT AND OBJECTIVE:**

Postoperative infections should be detected earlier. We investigated the efficacy of the National Nosocomial Infection Surveillance (NNIS) score, interleukin-6 (IL-6) and various acute-phase proteins for predicting postoperative infections.

**DESIGN AND SETTING:**

Case series study at the Júlio Müller University Hospital.

**METHODS:**

Thirty-two patients who underwent major gastrointestinal procedures between June 2004 and February 2005 were studied. The NNIS score and the evolution of serum IL-6 and various acute-phase proteins (C-reactive protein [CRP], albumin, prealbumin and transferrin) were correlated with postoperative infections and length of hospital stay (LOS).

**RESULTS:**

NNIS > 1 (p = 0.01) and low preoperative albumin (p = 0.02) significantly correlated with infection. IL-6 and CRP increased significantly more in patients with infections. Multivariate analysis showed greater risk of infection when NNIS > 1 (odds ratio, OR = 10.66; 95% confidence interval, CI: 1.1-102.0; p = 0.04); preoperative albumin < 3 g/dl (OR = 8.77; 95% CI: 1.13-67.86; p = 0.03); CRP > 30 mg/l on the second postoperative day (OR = 8.27; 95% CI: 1.05-64.79; p = 0.03) and > 12 mg/l on the fifth postoperative day (OR = 25.92; 95% CI: 2.17-332.71; p < 0.01); and IL-6 > 25 pg/ml on the fifth postoperative day (OR = 15.46; 95% CI: 1.19-230.30; p = 0.03). Longer LOS was associated with cancer, transferrin, IL-6 and albumin (p < 0.05).

**CONCLUSIONS:**

NNIS, albumin, CRP and IL-6 may be useful as predictive markers for postoperative infections. For predicting LOS, malignant condition, transferrin, albumin and IL-6 are useful.

## RESUMO

**CONTEXTO E OBJETIVO:**

Melhores resultados são esperados quando infecção pós-operatória é diagnosticada precocemente. Este estudo investigou a eficácia do escore NNIS (National Nosocomial Infection Surveil-lance score), interleucina-6 (IL-6) e proteínas de fase aguda na predição de infecções pós-operatórias.

**TIPO DE ESTUDO E LOCAL:**

Estudo tipo serie de casos na enfermaria de cirurgia geral do Hospital Universitário Julio Muller.

**MÉTODOS:**

32 pacientes submetidos a operações gastrointestinais de grande porte entre junho de 2004 e fevereiro de 2005 foram estudados. Correlacionou-se o escore NNIS, a evolução da IL-6 e varias proteínas de fase aguda (proteína C reativa [CRP], albumina, pré-albumina e transferrina) com a ocorrência de infecção e tempo de permanência hospitalar (LOS).

**RESULTADOS:**

O escore NNIS > 1 (p = 0.01) e a hipoalbuminemia pré-operatória (p = 00.02) significantemente correlacionaram com infecção. Ocorreu uma elevação maior de IL-6 e CRP nos pacientes com infecção. Análise multivariada mostrou maior risco de infecção em paciente com NNIS > 1 (razão de chances, RC = 10,6 [IC 95%, 1,1-102,2]; p = 0.04), albumina pré-operatória < 3 g/dl (RC = 8,77 [IC 95%, 1,13-67,86]; p = 0,03); CRP > 30 mg/l no 2^o^ (RC = 8,27 [IC 95%, 1,05-64,79]; p = 0,03) e > 12 mg/l no 5^o^ pós-operatória (RC = 25,92 [IC 95%, 2,17-332,71]; p < 0,01), e com IL-6 > 25 pg/ml no 5^o^ PO (RC = 15,46 [IC 95%, 1,19-230,30]; p = 0,03). LOS associou-se com câncer transferrina, IL-6 e albumina (p < 0,05).

**CONCLUSÃO:**

NNIS, albumina, CRP e IL-6 podem ser úteis como preditores de infecção pós-operatória. Na predição do LOS, a condição de doença maligna e a evolução de albumina, transferrina e IL-6 são úteis.

## INTRODUCTION

Infections play an important role in postoperative mortality rates.^1^ However, the main cause of mortality in such patients is multiple organ system dysfunction (MOSD) as a result of an exacerbated systemic inflammatory response, with or without infection.^1,2^ Preoperative identification of patients at risk of postoperative complications may have an impact on surgical management and enable treatment modifications so as to reduce morbid-mortality. Thus, the identification of variables of interest for predicting the risk of postoperative infections has become the main objective of several reports.^3-8^

Assessment of nutritional status and immune response may have prognostic value for such patients. It has been demonstrated that prior sensitization of the immune system may result in an inappropriate response to injury or infection. This sensitization is easily shown through the detection of components of the acute-phase response in the serum. There is evidence that exacerbation of the acute response is associated with a more aggressive inflammatory response.^9^ Thus, rather than diagnosing a nutritional deficit, it might be more important to assess its impact on the regulation of the immune system. This assessment can be carried out by detecting acute-phase proteins and cytokines.

There seems to be an association between preoperatively detected acute-phase response and the development of postoperative MOSD.^1,2^ Therefore, patients presenting acute-phase response during the preoperative period may be at risk. Studies focusing on the cause and functional characteristics of this preoperative or early postoperative sensitization could put forward therapeutic options for avoiding postoperative complications. Such assessments would enable individual identification of patients at risk, thereby restricting more vigorous assessments to a smaller number of patients.

Since the 1980s, several physiological scoring systems have been introduced for assessing patients with sepsis and MOSD, including the National Nosocomial Infections Surveillance (NNIS) score.^10^ This score assesses the intrinsic postoperative risk of infections at the site concerned and can predict infectious morbidity at other sites, such as pneumonia and urinary infections, and also overall mortality.^10^ The assessment of patients using these physiological scores makes it possible to estimate the risk for groups of patients, while identification of immune system activation by detecting inflammatory response markers allows individual assessment. The use of scoring systems such as NNIS^11^ allows stratification of the infection risk and, when this is added to analysis of inflammation markers during the postoperative period, they may be capable of selecting patients who are at greater risk of infectious morbidity and mortality, at lower cost and with greater efficacy.

On the other hand, there are still no ideal tests or markers for early prediction and identification of patients who are prone to septic complications. It is known that critically ill patients present an exacerbated systemic inflammatory response and that the start of such responses may precede the onset of clinical manifestations. Thus, the adoption of earlier strategies and therapeutic measures may benefit these patients. Among the various mediators of the systemic inflammatory response, C-reactive protein (CRP) has wide clinical application for confirming the existence of an inflammatory process, through its reasonable sensitivity and low cost. Recently, several studies have reported that the sensitivity and specificity of CRP in differentiating systemic inflammatory response syndrome (SIRS) and sepsis is around 60 to 90%.^12^ CRP is the prototype acute-phase protein, and its concentration rises sharply in response to various inflammatory stimuli, and particularly in response to interleukin-6 (IL-6).^13^

Previous studies have suggested that IL-6 serves as both a marker and a mediator for the severity of sepsis. Although IL-6 has both pro- and anti-inflammatory characteristics, when at high levels it correlates with the extent of the acute phase response and thus it has been considered to be an alarm indicator for the magnitude of the inflammatory response.^14,15^ The persistence of high IL-6 levels after the first postoperative day is a independent factor associated with septic postoperative complications.^16-18^

However, there are few studies on this issue, and no previous study has investigated the efficacy of the NNIS score and inflammatory markers at the same time. Furthermore, the definition of an ideal score for individual pre-operative prediction of septic complications and the creation of methods for early sepsis detection have not yet been drawn up, and efforts should continue to be made towards reaching these objectives.

## OBJECTIVE

The aim of this study was to investigate the efficacy of the NNIS score, IL-6 and various acute-phase proteins (CRP, albumin, prealbumin and transferrin) for predicting septic postoperative complications in patients who have undergone major surgical procedures in the digestive tract.

## METHODS

This study was carried out after gaining approval from the Research Ethics Committee of the Júlio Müller University Hospital (case number 146/CEP/HUJM/2004). Patients were included in the study after giving their informed consent.

### Patients

This was a case series study involving 32 consecutive patients, conducted at the Department of Surgery of the Júlio Müller University Hospital of the Federal University of Mato Grosso, Cuiabá, Brazil, between June 2004 and February 2005. The eligibility criteria for the study were that the subjects could be patients of either sex undergoing major elective surgery of the gastrointestinal tract with at least one intestinal anastomosis performed during the operation. The individual NNIS scores and the evolution of serum IL-6 and various acute-phase proteins (CRP, albumin, prealbumin and transferrin) were assessed in order to investigate associations with the occurrence of septic complications and length of hospital stay (LOS).

### Independent and outcome variables

The following clinical variables were recorded for all patients:

1. Preoperative variables: sex, age (above or below 50 years old), nutritional status (normal or malnourished, defined as the loss of 10% or more of habitual weight), body mass index (BMI), lymphocyte count/mm^3^, presence of comorbidities (diabetes mellitus, cardiac and/or renal insufficiency, chronic obstructive pulmonary disease, liver disease, or chronic diseases of the conjunctive tissue), malignant disease, and the American Society of Anesthesiologists (ASA) score (below/equal two or above two).^19^
2. Postoperative variables: NNIS score, which is a score ranging from 0 to 3 based on the presence (1 point) or absence (0 point) of three independent statuses: ASA score greater than two, operation classified as dirty, and duration of the operation longer than expected (more than three hours for esophagus, stomach, small intestine and colon operations, and more than four hours for hepatic, biliary and pancreatic procedures);^20^ and presence of infection, sepsis or septic shock according to the consensus of the American College of Chest Physicians/Society of Critical Care Medicine (ACCP/SCCM).^21^

Infectious morbidity and LOS were considered to be the main endpoints of the study. The NNIS score was categorized as high (scores 2 and 3) and low (scores 0 and 1). Serum IL-6, CRP, albumin, prealbumin and transferrin were assayed at the time of inducing anesthesia and on the second and fifth postoperative days.

### Definitions

The following definitions were adopted:

1. Infection of the surgical incision site: a) purulent drainage through the incision with or without laboratory confirmation; or b) isolation of organism from the wound culture or incision tissue culture and concomitant presence of at least one of the following infection signs and symptoms: pain or hypersensitivity, edema, hyperemia, or fever.
2. Intra-abdominal infection: a) purulent drainage through the drain; or b) isolation of organism obtained from abdominal cavity tissue or secretion; or c) abscess found during reoperation, histopathological examination or on image scan.^20^
3. Urinary tract infection: a) urine culture with 100,000 or more colony-forming units (CFU)/ml, with one or at most two bacterial species; or b) when two of the following parameters were present: fever, urination urgency, increased urination frequency, dysuria or suprapubic pain, in addition to one of the following parameters: pyuria, presence of nitrites in the urinary sediment, or positive bacterioscopy.^22^
4. Pneumonia: presence of new or progressive infiltrate, consolidation, cavitation or pleural effusion in chest X-ray, in addition to clinical manifestation.^22^
5. SIRS (systemic inflammatory response syndrome) and sepsis were defined according to the ACCP/SCCM^21^ consensus. SIRS was defined as a condition that included two or more of the following: temperature > 38° C or < 36° C; heart beats > 90/min; respiratory frequency > 20/min or PaCO_2_ < 32 mmHg and white cells > 12,000/mm^3^ or < 4,000 mm^3^, or > 10% young cells. Sepsis was defined if SIRS was associated with infection.

### Statistical method

The results were initially assessed by univariate analysis using either logistic or linear regression analysis, depending on the outcome variable, in an attempt to identify potential associations with infectious morbidity or LOS. Repeated-measurement analysis of variance (ANOVA) was used to analyze the evolution of all the acute-phase proteins and IL-6 for the presence or absence of infection using LOS as a covariable. Either linear or logistic multivariate regression analysis was used to determine the strength of association between independent variables and the outcome variables. Continuous variables (IL-6 and acute-phase proteins) were categorized to enter the logistic regression analysis. The cutoff used was the mean from the results observed among patients without infection, plus one standard deviation. The backward stepwise model was used for the regression construction. To evaluate whether the model fit the data, we used the goodness-of-fit test described by Hosmer and Lemeshow. Finally, validity parameters (sensitivity, specificity, positive and negative predictive values, and accuracy) were calculated for the variables that reached significance in the multivariate analysis. All the tests were performed using the Statistical Package for the Social Sciences (SPSS) for Windows 11.0. The results were expressed as means, odds ratios (OR) or β, and the respective 95% confidence intervals (95% CI), as appropriate. The statistical significance was set at 5% probability level (p < 0.05).

## RESULTS

The clinical characteristics of the patients studied and the procedures carried out on them are shown in Tables 1 and 2, respectively. Sixteen patients (50%) were considered malnourished. Malignancies were found in 11 (34.4%) patients. Eight (25%) patients needed additional procedures (n = 11) du ring the planned operation, to resolve intercurrences. All patients were included and thus there was no sample loss.

**Table 1.** Demographic and clinical characteristics of the 32 patients at baseline evaluation, before the gastrointestinal tract operation

Variable	measurement unity/type	n
Age	years: mean(range)	49 (18-72)
Sex	male/female	18/14
Body mass index	kg/m^2^: mean (range)	22.6 (16.0-46.8)
Malnutrition	yes: n (%)	16 (50%)
Lymphocytes	cells/mm^3^: mean (range)	1957 (610-3510)
American Society of Anesthesiologists score	n (%)	
	1	6 (18.7)
	2	18 (56.3)
	3	8 (25.0)
Malignant disease	n (%)	11 (34.4)
Presence of co-morbidity	yes: n (%)	15 (46.9)
Clinical diagnosis	^n (^%^)^	
	Biliary tract condition	7 (21.9)
	Peptic ulcer	6 (18.8)
	Benign esophageal condition	5 (15.7)
	Gastric cancer	3 (9.4)
	Periampullary cancer	3 (9.4)
	Colorectal cancer	3 (9.4)
	Megacolon	2 (6.2)
	Miscellaneous	3 (9.4)
**Total**		**32 100.0**

**Table 2.** Gastrointestinal operations performed and infectious morbidity incidence according to infection site

**Operation (n = 32)**	**n (%)**
Gastroenteral or enteroenteral derivation	8 (25.0)
Roux-en-Y biliodigestive derivation	6 (18.8)
Gastrectomy	4 (12.5)
Esophagectomy	3 (9.3)
Esophagocardioplasty	3 (9.3)
Colectomy	3 (9.3)
Duodenopancreatectomy	2 (6.3)
Transduodenal papillotomy	2 (6.3)
Low anterior resection of the rectum	1 (3.2)
**Infection site (n = 16)**	**n (%)**
Incision wound	10 (31.3)
Pneumonia	8 (25.0)
Urinary tract	2 (6.3)
Sepsis	4 (12.5)
Septic shock	3 (9.4)

The mortality rate was 6.3% (two cases), due to infection-related MOSD. Infections occurred in 16 patients (50%) who presented a total of 27 infection sites (Table 2). The most frequent infection site was the incision wound (31.3%, 10/32), followed by pneumonia (25%, 8/32). Six patients (18.7%) presented more than one infection site. The mean LOS was eight days (range: 1-60 days). Anastomotic dehiscence was observed in five patients (15.5%). Three noninfectious complications occurred in three patients (atelectasis, acute renal failure and deep vein thrombosis of an upper limb).

Among the clinical variables, only high NNIS score was significantly associated with infection (p = 0.01). LOS was associated with malignant disease and malnutrition. High NNIS score presented moderate association with LOS (β = 9.65; 95% CI: −0.29 to 19.6; p = 0.06) (Table 3).

**Table 3.** Univariate analysis by logistic and linear regression of preoperative and intraoperative variables, according to presence of postoperative infection or length of hospital stay (LOS)

**Postoperative infection**	**Odds ratio**	**95% CI**	**^p^**
Sex (male)	0.45	0.10 - 1.92	0.28
Age (> 50 years)	1.66	0.41 - 6.81	0.48
Nutritional status (malnutrition)	3.00	0.67 - 13.4	0.15
ASA (> 2)	1.96	0.38 - 10.16	0.41
NNIS (> 1)	6.60	1.40 - 31.05	0.01
Clinical diagnosis (cancer)	1.29	0.31 - 5.33	0.72
**Length of hospital stay (LOS)**	β	**95% CI**	^**p**^
Sex (male)	−6.45	−16.92 - 4.02	0.22
Age (> 50 years)	−4.92	−11.13 - 10.14	0.92
Nutritional status (malnutrition)	10.56	−0.40 - 20.73	0.04
ASA (> 2)	0.91	−11.27 - 13.10	0.88
NNIS (> 1)	9.65	−0.29 - 19.60	0.06
Clinical diagnosis (cancer)	11.11	1.20 - 21.03	0.03

*CI = confidence interval; ASA = American Society of Anesthesiologists score, NNIS = National Nosocomial Infection Surveillance score.*

### National Nosocomial Infection Surveillance scores

Seventeen patients (55.1%) presented NNIS scores of zero or one, and 15 (46.9%) scored greater than one. The infection rate was approximately six times greater among the patients scoring greater than one (Table 3): 5/17 (29.4%) versus 11/15 (73.3%); OR: 6.60; 95% CI: 1.40 to 31.05; p = 0.01.

### Acute-phase proteins and IL-6

**Figure 1:**
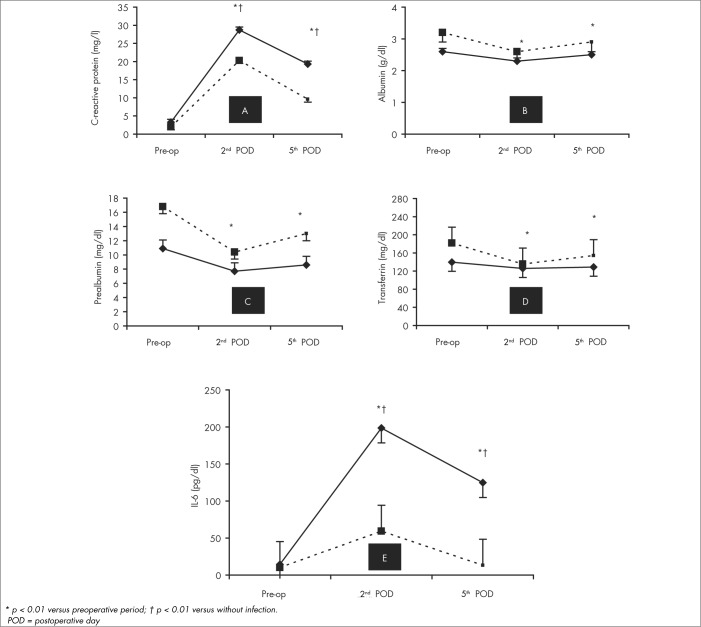
Evolution of various acute-phase proteins (A-D) and interleukin-6 (E) in patients with postoperative infections (solid lines) or without postoperative infections (dotted lines).

The descriptive data for the evolution of all the acute-phase proteins and IL-6, according to the presence or absence of infection and the LOS, can be seen in Table 4 and Figure 1.

**Table 4.** Evolution of the acute-phase proteins and interleukin-6 (IL-6) in operated patients with and without infection. Data are expressed as means and 95% confidence intervals, with repeated-measurement analysis of variance (ANOVA) using length of stay as a covariable

Variable	Infection		p (within groups)	p (between groups)
	Yes	No		
C-reactive protein (mg/l)				
Basal	3.3 (1.5-5.1)	2.0 (0.3-3.7)		
2^nd^ POD	28.7 (26.4-31.1)	20.3 (18.1-22.6)	< 0.001	< 0.001
5^th^ POD	19.3 (14.9-23.6	9.6 (5.4-13.7)		
Prealbumin (mg/dl)				
Basal	10.9 (6.9-15.0)	16.8 (12.8-20.9)		
2^nd^ POD	7.7 (5.8-9.7)	10.4 (8.5-12.4)	0.08	0.06
5^th^ POD	8.6 (5.3-11.9)	13.0 (9.7-16.3)		
Albumin (g/dl)				
Basal	2.5 (2.0-3.1)	3.2 (2.7-3.8)		
2^nd^ POD	2.2 (1.8-2.6)	2.6 (2.1-3.0)	0.14	0.20
5^th^ POD	2.4 (2.1-2.8)	2.9 (2.5-3.3)		
Transferrin (mg/dl)					
Basal	139.5 (105.7-173.4)	181.8 (147.9-215.7)		
2^nd^ POD	126.0 (107.3-144.7)	135.6 (116.9-154.3)	0.13	0.39
5^th^ POD	128.7 (102.9-154.6)	154.1 (128.2-179.9)		
IL-6 (pg/dl)					
Basal	13.2 (-3.9-30.1)	12.5 (5.4-19.6)		
2^nd^ POD	214.5 (77.7-351.3)	54.0 (20.8-87.1)	0.02	0.03
5^th^ POD	119.3 (3.9-234.7)	13.3 (-71-97)		

*POD = postoperative day.*

### C-reactive protein

There was no preoperative association between CRP levels and infection: OR = 1.50; 95% CI: 0.27-8.13; p = 0.64. There was a significant rise in CRP levels after the operation in all cases (p < 0.01). This increase in CRP was significantly greater in the patients with infection than without infection over time: second postoperative day, OR = 17.37, 95% CI: 2.43-124.18; fifth postoperative day, OR = 26.00, 95% CI: 2.98-226.97; p < 0.01. This was seen even when LOS was considered as a covariable (Table 4). On the fifth postoperative day, CRP levels decreased more significantly (p < 0.01) in patients without infection than others (Figure 1A).

LOS did not correlate with CRP, either preoperatively (β = 0.12; 95% CI: −1.36 to 1.61; p = 0.85) or on the second postoperative day (β = 0.29; 95% CI: −0.61 to 1.19; p = 0.52). However, there was a significant correlation between higher CRP levels on the fifth postoperative day and LOS (β = 0.86; [CI 95%: 0.34 to 1.19] p < 0.01). Thus, for each unit of elevation in CRP level, the LOS increased by 0.86 days on average.

### Albumin

Infection was approximately nine times more frequent in patients with preoperative albumin below 3 g/dl (OR = 9.10; 95% CI: 1.38-59.62; p = 0.02). The albumin level diminished over time (p < 0.01), with a significantly greater decrease in patients with infections than in others (p = 0.04). However, this difference did not correlate with infection when LOS was used as a covariable, either using repeated-measurement ANOVA or using logistic regression analysis: second postoperative day, OR = 0.92, 95% CI: 0.18-4.82, p = 0.98; and fifth postoperative day, OR = 1.18, 95% CI: 0.16-8.58, p = 0.87. These data are shown in Table 4 and Figure 1B.

Serum albumin significantly correlated with LOS at all three time points: preoperatively, β = −0.37, 95% CI: −11.97 to 0.08, p = 0.05; second postoperative day, β = −0.43, 95% CI: −18.04 to −2.04, p = 0.01; and fifth postoperative day, β = −0.57, 95% CI: −17.04 to −4.56, p = 0.01.

### Prealbumin

Similar preoperative prealbumin levels were presented by patients who acquired postoperative infections and by those who did not: OR = 2.50; 95% CI: 0.46-13.50; p = 0.29. Over time, there was a significant reduction in prealbumin levels in both groups (p < 0.01). However, this decrease was similar in the two groups when LOS was consider as a covariable, both by regression analysis (second postoperative day, OR = 2.14, 95% CI: 0.27-16.95, p = 0.47; and fifth postoperative day, OR = 3.64, 95% CI: 0.50-26.21, p = 0.20) and by ANOVA (Table 4 and Figure 1C).

There was a marginally significant correlation between serum prealbumin and LOS, both preoperatively (β = −0.66; 95% CI: −1.42 to −0.10; p = 0.08) and on the second postoperative day (β = −1.37; 95% CI: −2.87 to 0.11; p = 0.06). On the fifth postoperative day, however, the prealbumin levels correlated negatively with LOS. For each one-unit decrease in prealbumin, LOS increased by 0.78 days (β = −0.77; 95% CI: −1.53 to 0.00]; p = 0.05).

### Transferrin

At basal conditions, there was no difference in transferrin levels between those who acquired postoperative infections and those who did not (OR = 4.06; 95% CI: 0.63-26.13; p = 0.14). After the operation, there was a significant decrease in transferrin levels in the two groups (p = 0.01), but without any difference between the patients with or without posto perative infections (Figure 1D). When LOS was considered as a covariable, the comparisons between groups and within groups were not significantly different (Table 4).

The correlation between the preoperative transferrin level and LOS was marginally significant (OR = −0.34; 95% CI: −0.18 to 0.00; p = 0.07). On both the second and fifth postoperative days, the transferrin levels negatively correlated with LOS. In this context, the mean increases in transferrin of 10 units on the second postoperative day (OR = −0.48; 95% CI: −0.44 to −0.07; p < 0.01) and on the fifth postoperative day (OR = −0.44; 95% CI: −0.24 to −0.02; p = 0.01) decreased the LOS by approximately five and four days, respectively.

### Interleukin-6

At basal conditions, similar serum IL-6 levels were found in the two groups (OR = 4.33; 95% CI: 0.42-44.42; p = 0.21). The serum IL-6 levels increased in both groups on the second postoperative day (p < 0.001) and decreased thereafter. However, it was only in patients without infection that the IL-6 values returned to basal levels on day 5. Infection occurred around 8 to 20 times more frequently in patients with IL-6 higher than 101 pg/ml on the second postoperative day or higher than 26 pg/ml on the fifth postoperative day, respectively. IL-6 levels in patients with infection were significantly higher (p = 0.01) both on the second postoperative day (OR = 6.91; 95% CI: 1.06-45.22; p = 0.04) and on the fifth postoperative day (OR = 14.64; 95% CI: 1.90-113.04; p = 0.01), even when LOS entered as a covariable (Table 4 and Figure 1E).

There was no correlation between LOS and IL-6 values, either preoperatively (β = 0.00; 95% CI: −0.09 to 0.10; p = 0.96) or on the second postoperative day (β = 0.11; 95% CI: −0.02 to 0.03; p = 0.53). However, every ten-unit increase in serum IL-6 on the fifth postoperative day corresponded to an increase of approximately six days of hospitalization (β = 0.57; 95% CI: 0.02-0.09; p < 0.01).

### Multivariate analysis

In the multivariate logistic regression model, greater risk of infection was associated preoperatively with NNIS score greater than 1 (OR = 10.6; 95% CI: 1.1-102.0; p = 0.04) and preoperative albumin < 3 g/dl (OR = 8.77; 95% CI: 1.13-67.86; p = 0.03), even after adjusting for sex, age, malnutrition, ASA, cancer diagnosis and LOS. On the second postoperative day, CRP greater than 30 mg/l (OR = 8.27; 95% CI: 1.05-64.79; p = 0.03) in association with infection and IL-6 higher than 50 pg/ml was marginally significant (OR = 8.31; 95% CI: 0.81-84.50; p = 0.07). On the fifth postoperative day, CRP greater than 12 mg/l (OR = 25.92; 95% CI: 2.17-332.71; p < 0.01) and IL-6 greater than 25 pg/ml (OR = 15.46; 95% CI: 1.19-230.30; p = 0.03) were the only variables associated with infection (Table 5).

**Table 5.** Multivariate analysis by logistic and linear regression of perioperative variables, with statistical significance according to the presence of postoperative infection or length of hospital stay

**Postoperative infection**	**Odds ratio**	**95% CI**	**^p^**
NNIS (> 1)	10.60	1.10 to 102.00	0.04
Preoperative albumin < 3.0 g/dl	8.77	1.13 to 67.87	0.03
CRP > 30 mg/l on the 2^nd^ POD	8.27	1.05 to 64.79	0.03
CRP > 12 mg/l on the 5^th^ POD	25.92	2.17 to 332.71	< 0.01
IL-6 > 25 pg/ml on the 5^th^ POD	15.46	1.19 to 230.30	0.03
**Length of hospital stay (LOS)**	^β^	**95% CI**	^**p**^
Clinical diagnosis (cancer)	11.11	1.20 to 21.03	0.03
Transferrin on the 2^nd^ POD	−0.24	−0.46 to −0.02	0.03
Albumin on the 5^th^ POD	−8.13	−13.97 to −2.30	< 0.01
IL-6 on the 5^th^ POD	0.04	0.01 to 0.07	< 0.01

*CI = confidence interval; POD = postoperative day, NNIS = National Nosocomial Infection Surveillance score; CRP = C-reactive protein; IL-6 = interleukin 6.*

The validity parameters for these variables to predict postoperative infection can be seen in Table 6. The best prediction was found with CRP greater than 12 mg/l on the fifth postoperative day. Although showing low sensitivity, CRP greater than 30 mg/l on the second postoperative day was associated with both 100% specificity and 100% positive predictive value.

**Table 6.** Validity parameters for perioperative variables with statistical significance from multivariate analysis, for predicting postoperative infection

Variable	Sensitivity	Specificity	PPV	NPV	Accuracy
NNIS (> 1)	68.8	75.0	73.3	70.6	71.9
CRP > 30 mg/l on the 2^nd^ POD	31.2	100.0	100.0	57.7	64.5
Preoperative albumin < 3.0 g/dl	53.3	66.7	66.7	53.3	59.3
CRP > 12 mg/l on the 5^th^ POD	87.5	83.3	87.5	83.3	85.7
IL-6 > 25 pg/ml on the 5^th^ POD	81.2	75.0	81.2	75.0	78.6
IL-6 > 50 pg/ml on the 2^nd^ POD*	73.3	75.0	73.3	75.0	74.2

*NNIS = National Nosocomial Infection Surveillance score; POD = postoperative day; CRP = C-reactive protein; IL-6 = interleukin 6; PPV = positive predictive value; NPV = negative predictive value.*

* *= p = 0.08 in multivariate analysis.*

Among both the preoperative and the intraoperative variables, only the diagnosis of malignant disease condition was significantly associated with longer LOS (β = 11.11; 95% CI: 1.20-21.03; p = 0.03). On the second postoperative day, only the transferrin values were significantly associated with prolonged LOS (β = −0.24; 95% CI: −0.46 to −0.02; p = 0.03). On the fifth postoperative day, both IL-6 (β = 0.04; 95% CI: 0.01-0.07; p < 0.01) and albumin values (β = −8.13; 95% CI: −13.97 to −2.30; p < 0.01) correlated with prolonged LOS.

## DISCUSSION

The findings from this study have confirmed that both the acute-phase proteins and IL-6 become modified after the operative trauma. As a rule, there is a rapid increase in CRP and IL-6 and, conversely, a decrease in all other acute-phase proteins following the operation. The results also showed that these modifications differ according to whether patients acquire postoperative infection or not. These findings are in line with some previous studies that have already shown that acute-stage proteins and IL-6 present a relationship with infectious complications in the postoperative period.^23-28^

Our data suggest that the NNIS index and preoperative albumin level are important predictive factors for perioperative infection. Indeed, except for albumin, no other biochemical marker measured during the operation was predictive of infection in the postoperative period in the light of multivariate analysis. However, both NNIS and preoperative albumin had low sensitivity for predicting postoperative infections. These findings are important, because no other previous study has correlated NNIS scores with acute-phase proteins or cytokines.

In the early postoperative period, IL-6 and most of the acute-phase proteins were correlated with postoperative infection by means of univariate analysis. However, when the data were analyzed using multivariate logistic regression, CRP on the second day and again CRP and IL-6 on the fifth day were the only predictive factors for postoperative infection. When these results were seen in the light of the accuracy and predictive value indices, CRP again reaffirmed its position as a good predictor of infection on both the second and the fifth postoperative days. Taking all this in account, the data suggest that CRP might be the most important predictive factor for postoperative infection. However, these results should be considered with caution, because the power analysis for some parameters, such as for transferrin, was very low. Nevertheless, the power analysis for both CRP and IL-6 was greater than 90%. Within this context, there is some controversy in the literature and a few studies have shown IL-6 to be more efficient than CRP.^27-29^

Both high NNIS scores and the presence of malignant disease were clinical factors significantly associated with longer LOS by univariate analysis. However, in the multivariate analyses, only malignant conditions were associated with prolonged hospitalization. The nutritional state was only marginally predictive in this analysis. Again, because of the small number of cases in this study, these data should be considered with caution, because previous studies have demonstrated the value of nutritional status assessment in predicting complications and LOS.^9,24,25^ The acute-phase proteins and IL-6 did not correlate with longer LOS either during the operation or during the early postoperative course. However, the data from the fifth postoperative day strongly correlated IL-6 and albumin with longer LOS. In this context, maintenance of a low level of albumin or a high level of IL-6 by the fifth postoperative day was found to be a predictive factor for longer LOS. In the same way, persistence of low serum levels of CRP, prealbumin or transferrin was prognostic for longer hospitalization. The multivariate analysis showed, however, that transferrin on the second postoperative day and both albumin and IL-6 on the fifth postoperative day were the most powerful variables associated with LOS. These findings suggest that it is possible to create a protocol to show which are the best markers for predicting LOS, according to the postoperative day. Thus, these results call for further investigations on this issue.

Another important finding from this study was the confirmation that the immune-inflammatory response evaluation may be of more importance than simple diagnosis of the nutritional status, for evaluating the risk of postoperative morbidity. Our data are therefore in agreement with the two-hit theory of MOSD and, thus, reinforce the importance of investigating acute-phase proteins or cytokines during the perioperative period in patients who will undergo major operations.^3-8,17,23,26^

Patients who are candidates for major operations on the abdominal cavity deserve attention because various factors, including previous bacterial infections, malnutrition and malignancies, for instance, may primarily trigger an immune system response.^23^ Patients who are more prone to complications are also more reactive to operative trauma than others with a normal immune response. In this context, a smaller bacterial contamination may provoke an intense immune-inflammatory response with greater release of cytokines and greater acute-phase protein response. The common pathway for such cases is their predisposition to postoperative infections, intense SIRS and MOSD.^23^

## CONCLUSIONS

In summary, the overall results from this study have clearly shown that the acute-phase response does exist and is greater in cases of postoperative infections. Moreover, our data suggest that changes detected early in the evolution of either acute-phase proteins or IL-6 may alert the surgeon to the development of insidious infection that may not have been clinically evident until then. This is crucial and could change the management for many patients. Out of all the variables studied, CRP was the most important acute-phase protein for predicting postoperative infection. Another possible conclusion is that NNIS scores are an important instrument for predicting posto perative infections. Although NNIS scores, in association with other acute-phase proteins such as CRP, albumin and prealbumin, and with IL-6, were not the most effective tool, they may enable more accurate prognoses for postoperative infection. With regard to predicting longer LOS, it was found that malignant condition, transferrin, albumin and IL-6 were generally useful.
